# Editorial: Molecular and cellular pathways leading to mitochondrial dysfunction and neurodegeneration: Lessons from *in vivo* models

**DOI:** 10.3389/fnins.2022.1006100

**Published:** 2022-09-07

**Authors:** Shabab B. Hannan, Alvaro Sanchez-Martinez, Gloria Brea-Calvo, Aurora Gomez-Duran, Juan A. Navarro

**Affiliations:** ^1^German Center for Neurodegenerative Diseases, Hertie Institute of Clinical Brain Research, University of Tübingen, Tübingen, Germany; ^2^MRC Mitochondrial Biology Unit, University of Cambridge, Cambridge, United Kingdom; ^3^Andalusian Centre of Cell Biology, University Pablo de Olavide and CIBERER (ISCIII), Seville, Spain; ^4^MitoPhenomics Lab, Biological Research Center Margarita Salas-CSIC, Madrid, Spain; ^5^INCLIVA Biomedical Research Institute, Valencia, Spain

**Keywords:** mitochondria, neurodegenerative disease, mitophagy, mitochondrial dynamics, MAMs, lysosome, animal models

Mitochondria are highly dynamic organelles that, beyond ATP synthesis, are involved in diverse cellular processes, including reactive oxygen species (ROS) production, lipid metabolism, calcium buffering, and cell death. Neurons are particularly dependent on mitochondria, and consequently, impairment in mitochondrial function renders them susceptible to diverse insults. Thus, an increasing number of genetic studies continue to implicate mutations in mitochondrial associated genes to common and rare neurodegenerative disorders, making these organelles a promising therapeutic target (Russell et al., 2020). In this Research Topic, experts in the field contributed original research in *in vivo* models and mammalian culture systems as well as timely reviews on emerging new aspects of mitochondrial biology in health and neurological diseases. Literature reviews and original research articles highlight mitochondrial involvement in several neurodegenerative diseases including Alzheimer's disease (AD), Parkinson's disease (PD), Huntington's disease, frontotemporal dementia (FTD), amyotrophic lateral sclerosis (ALS), optic atrophy (OA), hereditary spastic paraplegia (HSP) and HIV-associated neurocognitive disorders (HAND).

Cells display several quality control mechanisms to safeguard mitochondrial integrity such as the unfolded protein response (UPR), mitochondrial dynamics and their degradation by mitophagy. In this topic, Hu et al. reviewed these processes and summarized their alterations in several neurodegenerative diseases. The dissection of the genetic pathways controlling them paves the way for the identification of therapeutic avenues that might improve mitochondrial function and concomitantly patient wellbeing. Activation of the UPR response is common in several proteinopathies including ALS and ALS-related disorders. On that note, Benson et al. comprehensively reviewed the pathophysiology of these pathologies aiming to identify overlapping pathological hallmarks that might indicate common disease mechanisms. Besides the classical alterations in the cellular degradation systems, authors also discussed evidence for RNA metabolism and mitochondrial dysfunction that have recently emerged as core elements in the development and progression of these neurological disorders. Complementarily, Anoar et al. addressed these neuropathies (ALS and FTD) providing a fruitful viewpoint on findings, particularly from *Drosophila* models, that have progressed our understanding of mitochondrial involvement in the pathogenesis of these two interconnected diseases, characterized by declining motor and cognitive functions.

Mitochondrial function is tightly coordinated by dynamic capacity of these organelles comprising a plethora of processes ranging from formation to degradation as well as organelle plasticity (Chan, 2020). These processes are regulated by factors encoded by nuclear and mitochondrial genomes and mutations in these genes have been linked to several diseases including neurodegenerative diseases. In this Research Topic, Strachan et al. comprehensively reviewed the human molecular genetics of autosomal OA, caused by degeneration of retinal ganglion cells (RGCs) due to defects in genes involved in diverse functions including mitochondrial dynamics, cellular respiration, mitochondrial DNA replication and fatty acid synthesis. Evaluation of several *in vivo* models provides in-depth insights of mitochondrial involvement in pathophysiology of diseases and emphasizes their involvement in pathways leading to susceptibility of RGCs. Pijuan et al. extended the analysis to several genes involved in mitochondrial dynamics (*DRP1, GDAP1, OPA1* or *MFN2)* and other mitochondrial genes *(FXN, MED13* and *CHKB)* using fibroblasts from patients to study the consequences of mutations in these genes on the mitochondrial network morphology, oxidative stress and membrane potential. Interestingly, they found a pathophysiological association between mitochondria and lysosomes through their contact sites highlighting a crosstalk between these organelles with a critical role for *MFN2* and *GDAP1* (Cantarero et al., 2020).

Furthermore, a crucial factor of cell function is the ability to maintain organelle communication (Mottis et al., 2019). In our collection, mitochondrial communication with other organelles in neurological diseases has been highlighted by Proulx et al. In this comprehensive review, the authors provide evidence of the disruption of mitochondria associated ER membranes (MAM) *in vitro* and *in vivo* models of AD, PD and ALS. MAMs are crucial inter-organellar structures between ER and mitochondria and act as essential platforms to coordinate diverse cellular processes including mitochondrial dynamics and bioenergetics, calcium and lipid homeostasis, autophagy, apoptosis, inflammation, and intracellular stress responses (Area-Gomez et al., 2018; Gómez-Suaga et al., 2018; Lau et al., 2018). Remarkably, Proulx et al. also discussed implications of MAMs in HAND, a feature that has not yet been explored in neuropathology. The authors stress that to identify potential targets for therapeutic interventions, a deeper comprehension of the processes governing changes in MAMs in a particular cell type and/or disease is essential.

While most axonal mitochondria are stationary, some are in transit between cell body and distal synapses (Misgeld and Schwarz, 2017). Mitochondrial transport along axons is critical for replenishment of distal neuronal segments of highly polarized neurons. Importantly, disruption of mitochondrial trafficking along axons is a common feature in neurodegenerative disorders. In particular, it is the largest motor neurons, innervating muscles in lower limbs that are most vulnerable to disruption in axonal transport as manifested in HSP (Maday et al., 2014). Güner et al. investigated mitochondrial pathology associated with spastic paraplegia 11 (SPG11) pathogenic variants in induced pluripotent stem cells (iPSC)-derived motor neurons (MNs) from patients and healthy donors. Interestingly, Güner et al. report compartment-specific morphological defects of mitochondria in neurites of iPSCs-derived MNs, but not within the soma. Furthermore, mitochondria of SPG11-defective MNs showed impaired membrane potential and reduced anterograde transport. While the mechanism behind such compartment-specific defects is unclear, this study points to different roles of various neuronal compartments in mitochondrial life cycle and quality control.

Mitochondrial function is also critical for the transmission of information and nerve impulses at the synaptic cleft. In support of this, Mallik and Frank reported a novel role of mitochondrial complex I component ND-20L in the maintenance of synaptic structure and function using the *Drosophila melanogaster* neuromuscular junction (NMJ) as a model system. Utilizing an electrophysiological screen involving knock-down of human neurological and muscle disease-related genes in a tissue-specific manner, the authors found that loss of ND-20L impaired synaptic transmission and altered NMJ morphology.

In conclusion, this compendium of information will help the readership navigate the literature by providing an overview of some of the most relevant research themes in mitochondrial biology in the context of neurodegenerative diseases (Figure 1). In addition, at the translational level, we hope this will grant an insight into potential therapeutic approaches that could be used to treat mitochondriopathies.

**Figure 1:**
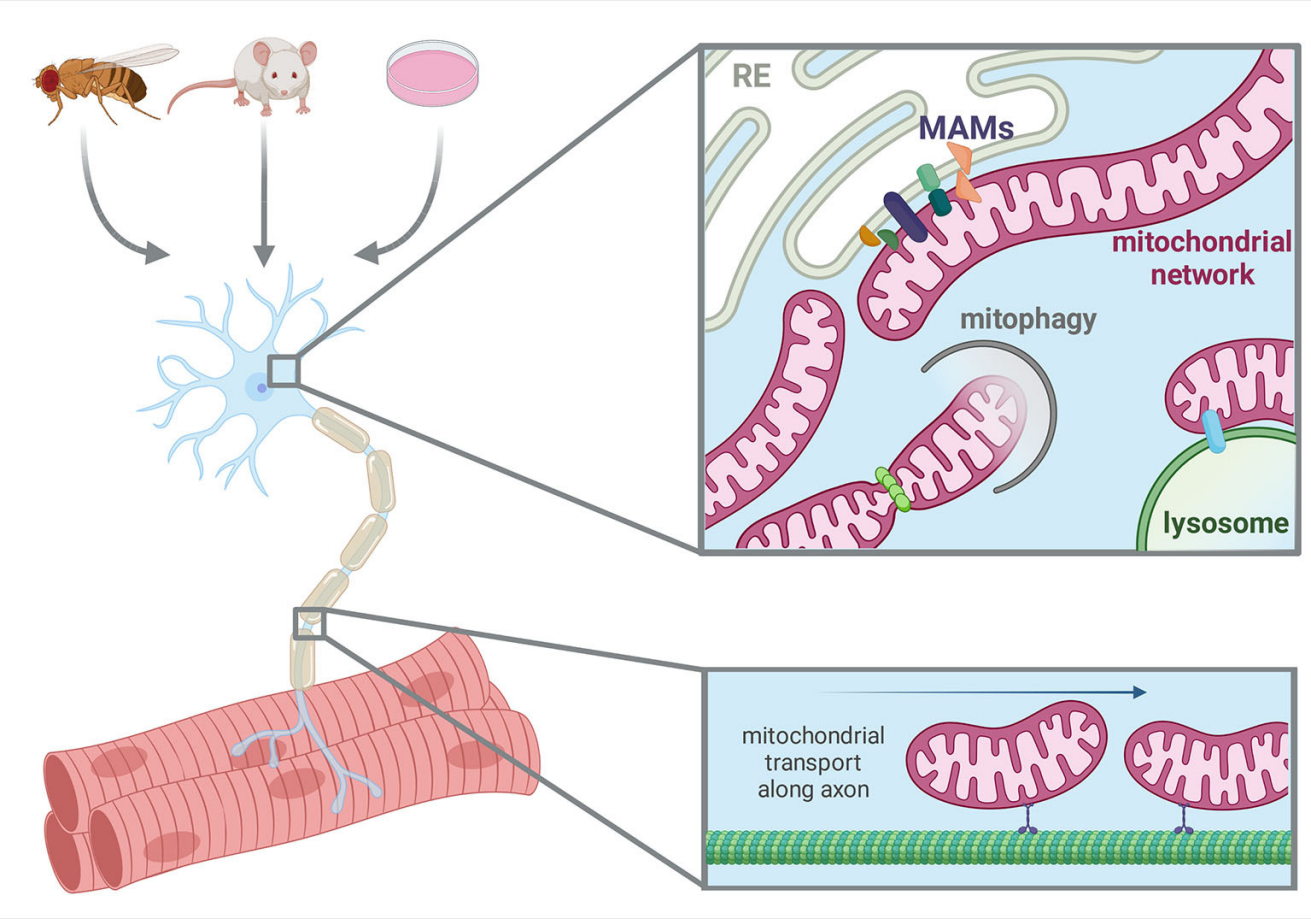
Model systems utilized and aspects of neuronal mitochondrial biology in this Research Topic. Created with BioRender.com.

## Author contributions

All authors listed have made a substantial, direct, and intellectual contribution to the work and approved it for publication.

## Conflict of interest

The authors declare that the research was conducted in the absence of any commercial or financial relationships that could be construed as a potential conflict of interest.

## Publisher's note

All claims expressed in this article are solely those of the authors and do not necessarily represent those of their affiliated organizations, or those of the publisher, the editors and the reviewers. Any product that may be evaluated in this article, or claim that may be made by its manufacturer, is not guaranteed or endorsed by the publisher.
